# Supplementary material for: Sirtuin1-Mediated Deacetylation of Hypothalamic TTF-1 Contributes to the Energy Deficiency Response
Source: Int J Mol Sci. 2023 Aug 7;24(15):12530. doi: 10.3390/ijms241512530 (PMC10419861; doi:10.3390/ijms241512530)
Supplement: Supplementary file 1 [file ijms-24-12530-s001.zip › ijms-2542235-supplementary.pdf]

Supplementary Materials

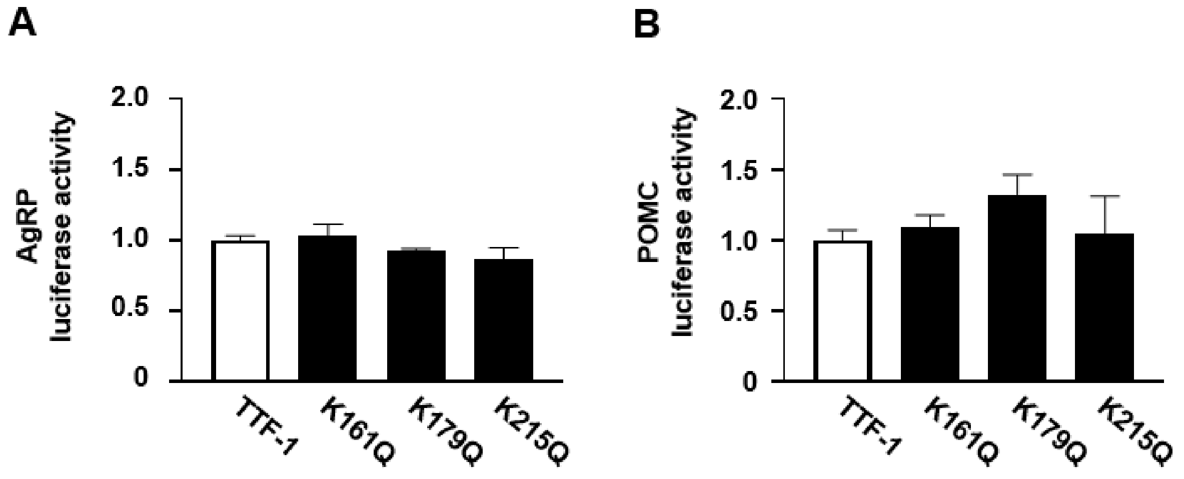

Supplementary Figure S1. *Effect of TTF-1 mutants on AgRP and POMC promoter activity.* AgRP (A) and POMC (B) promoter-luciferase activities were measured in mHypoA cells with transfection of expression vectors for TTF-1 and mutant TTF-1 (K161Q, K179Q, and K215Q) (n = 4/group). All data represent arbitrary units.

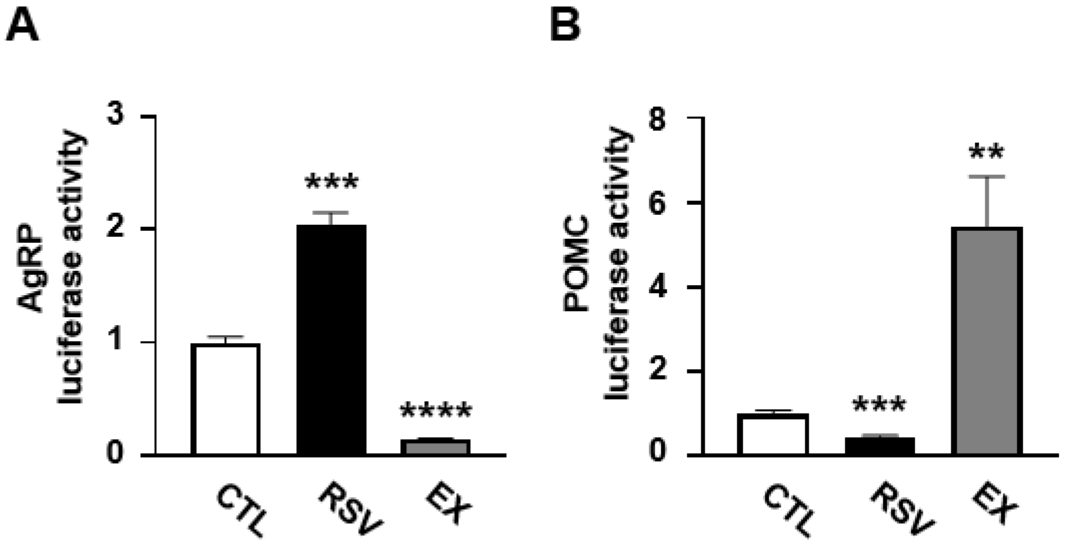

Supplementary Figure S2. *Effect of resveratrol and EX527 on AgRP and POMC promoter activity.* To validate the effects of a Sirt1 activator, resveratrol (RSV), and a Sirt1 inhibitor, EX527 (EX), on the expression of AgRP and POMC genes, the promoter activities of AgRP (A) and POMC (B) were measured using luciferase assays in the mHypoA cells after treatment for 24 h with RSV or EX (n = 4/group). **$P < 0.01$, ***$P < 0.001$, ****$P < 0.0001$. All arbitrary units.

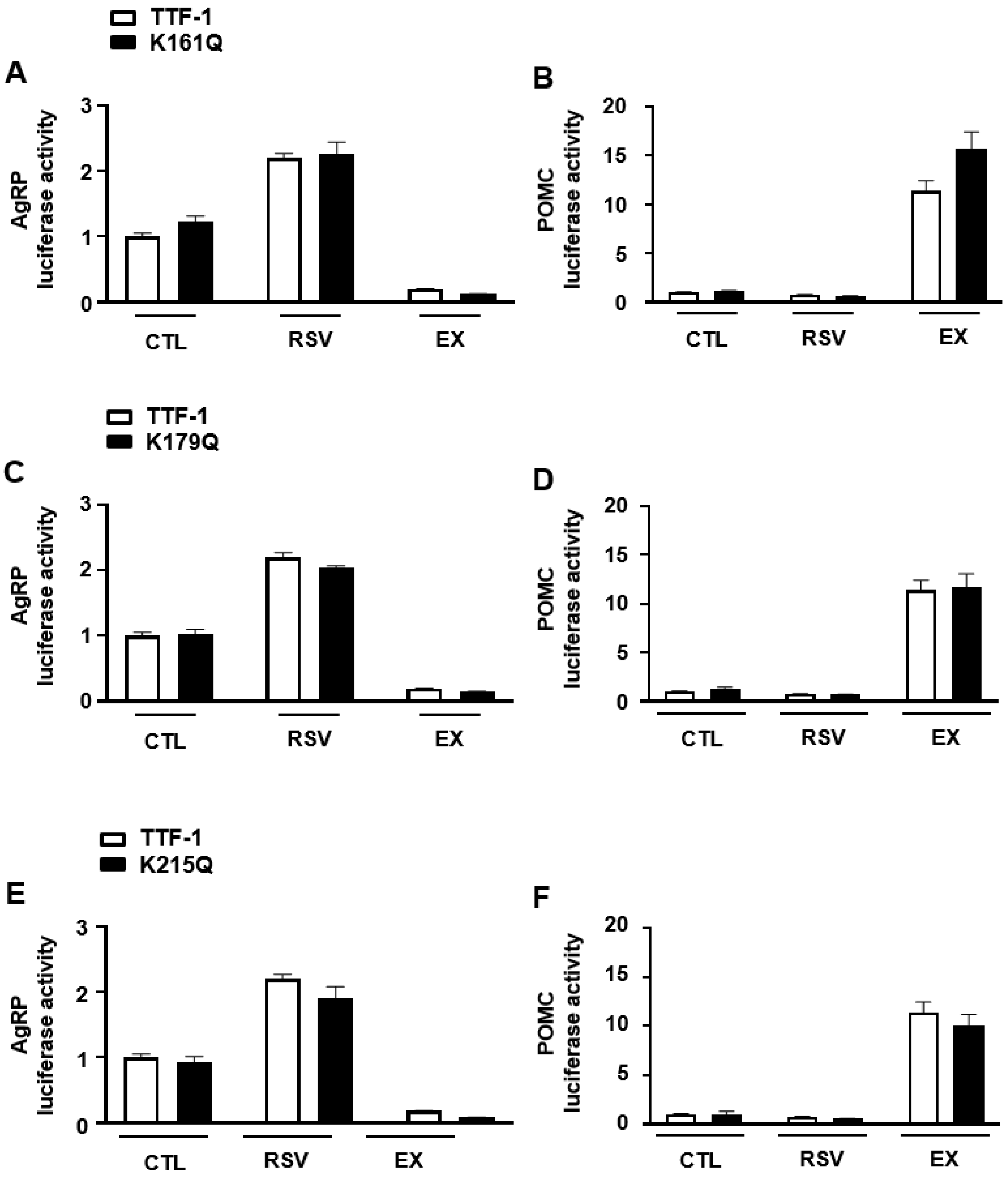

Supplementary Figure S3. *Effect of resveratrol and EX527 on the action of TTF-1 mutants on the AgRP and POMC promoter activity.* To verify the effect of a Sirt1 activator, resveratrol (RSV), and a Sirt1 inhibitor, EX527 (EX), on the action of TTF-1 mutants on the AgRP and POMC promoter activities, mHypoA cells were transfected with vectors for AgRP and POMC promoter-luciferase and expression vectors for TTF-1 and its mutants (K161Q, K179Q, and K215Q). The cells were treated for 24 h with either RSV or EX and the resulting luciferase activities for AgRP (A, C, E) and POMC (B, D, F) promoters were measured (n = 4/group). No significance was found between groups. All data represent arbitrary units.
